# A comparative study of prone split-leg position and lithotomy position in posterior uterine myomectomy by transvaginal natural orifice transluminal endoscopic surgery

**DOI:** 10.1186/s12905-025-03709-z

**Published:** 2025-04-12

**Authors:** Yayu Zhou, Yonghong Lin, Dingyu Xu, Li He, Lu Huang

**Affiliations:** Department of Gynecology, School of Medicine, Chengdu Women’s and Children’s Central Hospital, University of Electronic Science and Technology of China, No. 1617, Riyue Avenue, Qingyang District, Chengdu, Sichuan 610000 China

**Keywords:** Transvaginal natural orifice transluminal endoscopic surgery, Myomectomy, Prone split-leg position, Lithotomy position, Comparative study

## Abstract

**Background:**

To assess and compare the efficiency and outcomes between prone split-leg position and lithotomy position in posterior uterine myomectomy by transvaginal natural orifice transluminal endoscopic surgery (vNOTES).

**Methods:**

33 patients with posterior uterine myomectomy by vNOTES in the prone split-leg position and 15 patients in the lithotomy position were retrospectively recruited. Important baseline characteristics and outcome parameters such as age, body mass index, volume of myoma, delivery mode, hospital length, intraoperative blood loss, hemoglobin values before and 72 h after operation, VAS score, operation time and operation preparation time were compared between two patient groups.

**Results:**

The operation time of the prone split-leg position group was significantly shorter than that of the lithotomy position group (*P* < 0.05), but the operation preparation time of the prone split-leg position group was longer than that of the lithotomy position group (*P* < 0.05). No significant difference was found in other indicators between the two patient groups.

**Conclusions:**

Our study suggests the potential application of the prone split-leg position in posterior uterine myomectomy by vNOTES.

**Supplementary Information:**

The online version contains supplementary material available at 10.1186/s12905-025-03709-z.

## Background

Natural orifice transluminal endoscopic surgery (NOTES) is an evolving minimally invasive modality that uses the natural orifice of the human body, such as perforation of the oral cavity, anus, vagina, or urethra and viscera, as the surgical channel for endoscopic access to the abdominal cavity to reach the target tissue for operation [1]. This surgical technique obviates the necessity for abdominal incisions, thus mitigating incision-related complications including postoperative abdominal wall pain, incision infection and hernia, and achieving higher cosmetic requirements. NOTES has gained significant adoption in gynecology, particularly in surgeries such as adnexal surgery, hysterectomy, and myomectomy [2].

Its transvaginal iteration, known as transvaginal NOTES (vNOTES), has experienced significant adoption in gynecological surgery, such as adnexal surgery, hysterectomy, and myomectomy [2]. However, the application of vNOTES to posterior uterine fibroids is relatively limited due to the restrictions of vision and surgical operating space. Therefore, it is necessary to identify a more suitable surgical position for myomectomy of the posterior uterine wall of the uterus by vNOTES. The lithotomy position is commonly used in gynecological surgery [3, 4], however, it presents challenges when addressing myomas situated on the posterior uterine wall. Employing diverse instruments to operate within the vesicorectal depression in this posture is akin to conducting surgery on a “ceiling”, with poor vision and limited manipulation space, thus increasing the difficulty of surgery.

Recent studies in gynecology have highlighted the growing popularity of the vNOTES approach in various gynecological procedures. For instance, one study compared vNOTES with conventional laparoscopy in bilateral salpingectomy for permanent female sterilization, demonstrating that vNOTES was associated with lower postoperative pain, reduced analgesic use, and higher patient satisfaction [5]. In a similar vein, another evaluation of vNOTES in gynecological emergencies, including ectopic pregnancy and ovarian torsion, found that vNOTES resulted in significantly shorter surgery times, reduced postoperative pain, and shorter hospital stays compared to conventional laparoscopy [6]. Moreover, further research has reinforced the feasibility and safety of vNOTES for treating benign gynecological conditions, confirming its potential as a minimally invasive alternative to traditional laparoscopic methods [7]. Building on this, a study exploring the ‘vNOTES first’ strategy for benign gynecological surgeries revealed that it led to shorter hospital stays, lower pain scores, and improved patient satisfaction [8]. Additionally, evidence supporting vNOTES in treating pelvic organ prolapse and myomectomy has shown excellent functional and anatomical outcomes [9]. Finally, the successful use of vNOTES in a pregnant woman for managing acute abdominal pain further emphasized its safety and lack of long-term negative effects on pregnancy and vaginal birth [10].

To enlarge the surgical field of vision, shorten the operation time, and reduce the use of anesthetic drugs, our surgical team has tried to use the prone split-leg position as the surgical position for myomectomy of the posterior wall of the uterus by vNOTES, and we assessed the potential differences between lithotomy position and prone split-leg position in myomectomy of the posterior wall of the uterus by vNOTES. In this study, we aimed to assess and compare the important baseline characteristics and outcome parameters such as age, body mass index, volume of myoma, delivery mode, hospital length, intraoperative blood loss, hemoglobin values before and 72 h after operation, VAS score, operation time and operation preparation time between prone split-leg position and lithotomy position in posterior uterine myomectomy by vNOTES, thus providing new evidence in clinical practice for patients with posterior uterine myomectomy.

## Methods

### Baseline characteristics

This is a single-center, retrospective study. Patients who underwent myomectomy of the posterior wall of the uterus by vNOTES in Chengdu Women’ s and Children’s Central Hospital from January 2019 to August 2022 were included in this study. As this is a retrospective study, patients were not randomized in the traditional sense. Instead, they were grouped based on their clinical treatment choice: those who underwent myomectomy in the prone split-leg position (PSP group) and those who underwent myomectomy in the lithotomy position (LP group). All the patients were diagnosed as uterine leiomyoma by postoperative pathological examination, and preoperative and intraoperative imaging confirmed single uterine leiomyoma located in the posterior wall of the uterus. This study was approved by the ethics committee of Chengdu Women’ s and Children’ s Central Hospital.

### Inclusion and exclusion criteria

The inclusion criteria of patient collection in this study were as follows: (1) Women over 18 years old with a clear surgical pointer; (2) Stable vital signs allow for laparoscopic surgery; (3) patients can understand the research program and are willing to participate in this study, providing written informed consent. And the exclusion criteria were shown as follows: (1) acute infection stage, preoperative deep venous thrombosis or hypercoagulability, fasting blood sugar > 11.1 mmol/l, blood pressure > 160/100 mm Hg, liver and kidney dysfunction, mental illness and other surgical contraindications; (2) a history of rectal surgery, suspected of rectovaginal septum endometriosis, tumors, or severe adhesions; (3) virginity; (4) pregnancy.

### Grouping and surgery

**Fig. 1:**
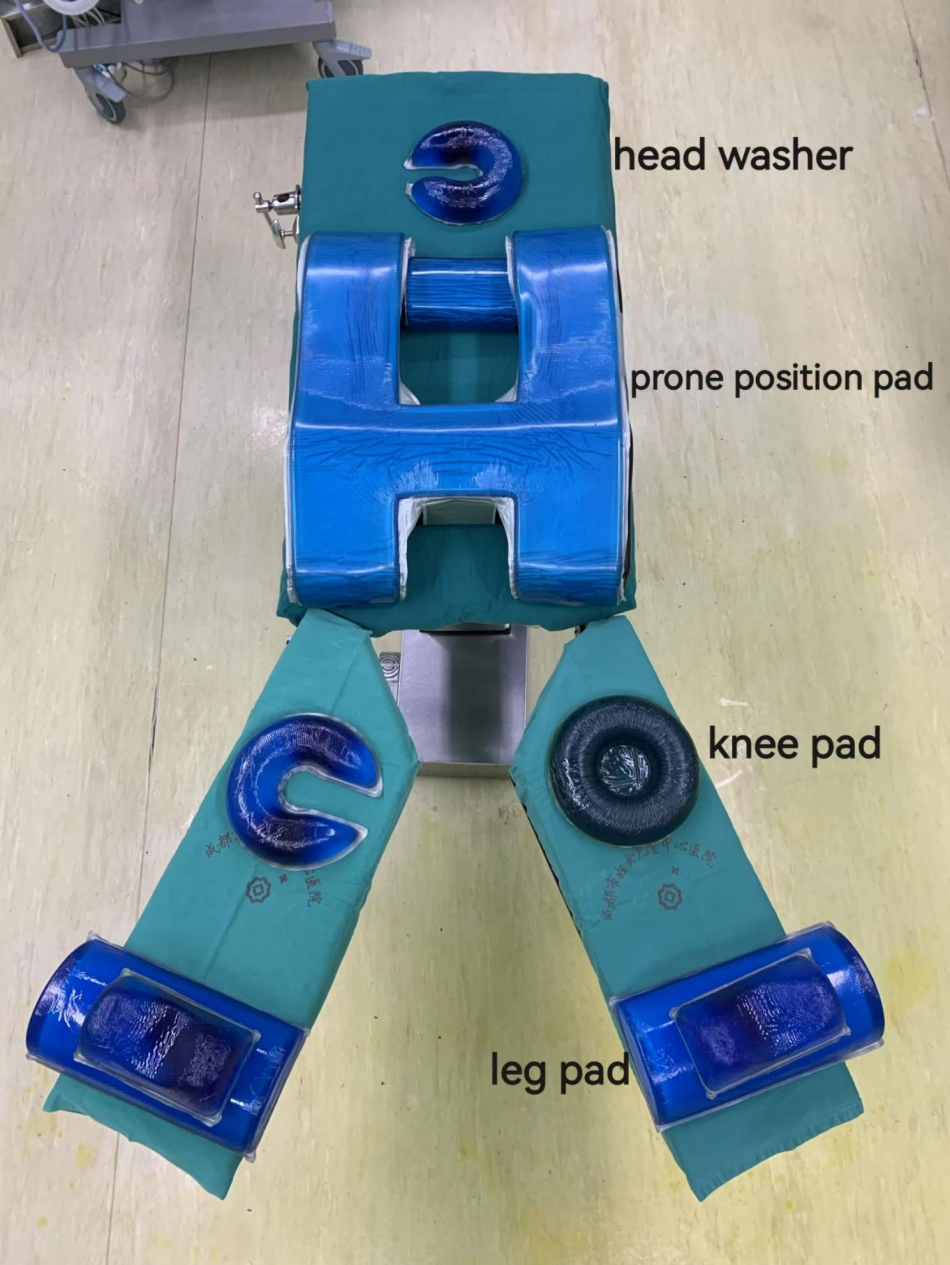
Preparation of instruments. This image shows the preparation of essential surgical tools and equipment prior to surgery, ensuring all necessary tools are ready for the procedure

The patients were divided into PSP group and LP group. (1) PSP group: After general anesthesia with endotracheal intubation in the supine position, the patient was placed in the prone split-leg position, with the legs separated and the head deviated to one side to facilitate endotracheal tube patency. A gel pad was placed on the operating table before the patient was placed in the prone split-leg position to prevent pressure ulcers. CO2 pneumoperitoneum was performed, and the intra-abdominal pressure was carefully controlled and maintained below 14 mmHg to ensure proper abdominal expansion, which is essential for optimal visualization and surgical access (Fig. 1). (2) After general anesthesia with endotracheal intubation in the supine position, the patient was placed in the LP. Upon achieving the requisite positioning, the surgical procedure was executed for both cohorts of patients. The sequence of steps followed was as follows: First, the posterior vaginal vault was incised in the Douglas pouch, and PORT (a VONTES dedicated device) was placed. CO_2_ pneumoperitoneum was accessed, and the intra-abdominal pressure was kept below 14 mmHg. After the location of the myoma was determined, the pituitrin 6U was injected into the myometrium of the uterus and the protrusion site was incised with a monopolar hook. Myomectomy was performed in the same way as conventional laparoscopic surgery. The mass was then bagged and cut into pieces if necessary. Last, the vaginal vault incision was sutured. All surgical procedures were performed by the same surgical team.

**Fig. 2:**
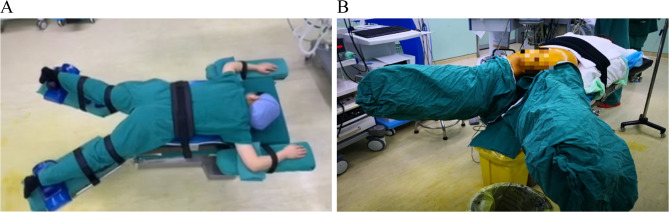
Positioning of the patient. (**A**) Prone position. This image shows the patient set up in the prone split-leg position, providing optimal visibility and working space for the surgery. (**B**) Detailed setup of the prone split-leg position. This image illustrates the detailed setup, highlighting support pads and head alignment to ensure patient stability and comfort

**Fig. 3:**
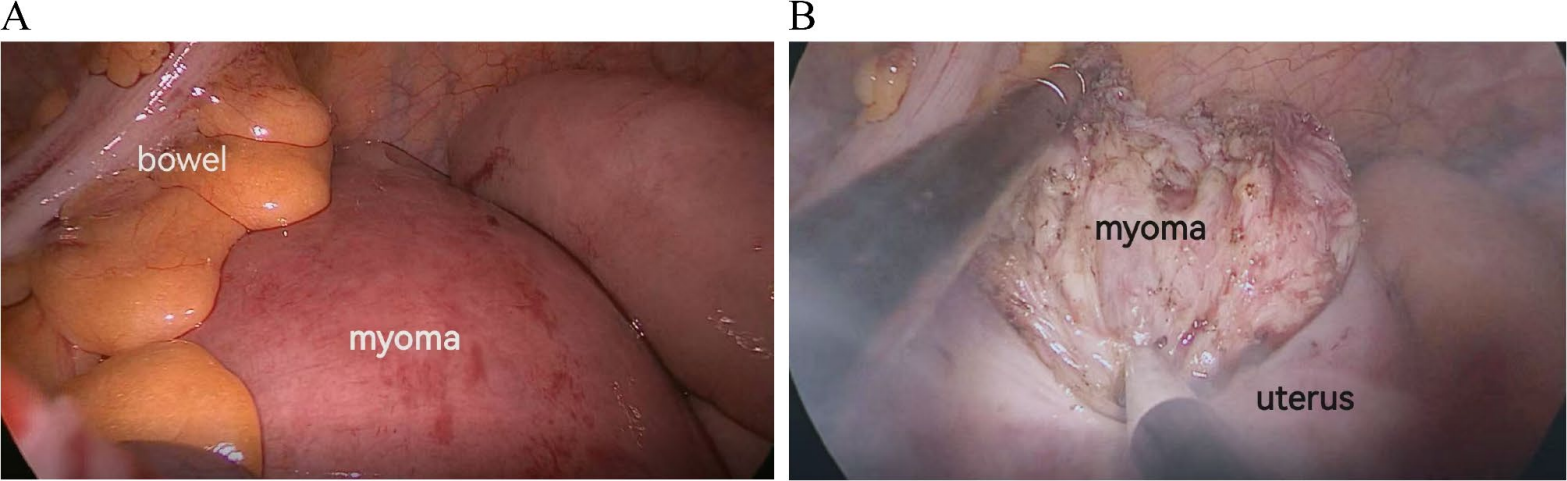
Intraoperative process. (**A**) Posterior uterine myoma. This intraoperative image shows the posterior uterine myoma, with clear markings indicating the location of the fibroid and surrounding structures, providing a reference for the myomectomy procedure. (**B**) Enucleation of the myoma. This image demonstrates the enucleation of the myoma, showing how the fibroid is dissected and separated from the uterine wall

To ensure optimal surgical access and patient stability, Fig. 2 (A) shows the patient in the prone split-leg position, which allows for clear visibility during posterior uterine myomectomy. Figure 2 (B) further illustrates the detailed setup of this position, emphasizing the use of support pads and proper head alignment to maintain the patient’s stability and comfort throughout the procedure. Intraoperatively, Fig. 3 (A) clearly shows the posterior uterine myoma, highlighting its location and the surrounding structures, which aids in precise dissection. Figure 3 (B) demonstrates the enucleation process of the myoma, depicting how the fibroid is separated from the uterine wall and prepared for removal.

To preempt potential infection, 1 g cefmetazole was intravenously administered within 30 min preceding the procedure, followed by additional doses at 12-hour intervals. All patients received a patient-controlled analgesia pump to relieve postoperative pain. Before discharge, three criteria necessitated fulfillment including: (1) normal body temperature for at least 24 h; (2) no surgical complications; (3) normal blood routine test results [11].

### Indicators

The indicators including baseline characteristics and outcome measures were analyzed and compared between PSP group and LP group. The baseline indicators include age, body mass index (BMI), volume of myoma and delivery mode, and the outcome indicators contained hospital length, intraoperative blood loss, hemoglobin values before and 72 h after the operation, VAS score, operation time and operation preparation time. Among these, preoperative preparation time refers to the period from the start of anesthesia to the start of surgery; the volume of myoma was calculated with the ellipsoidal formula according to the size measured by ultrasound: L × W × D × 0.523, where L = length, W = width, and D = depth [11]; the VAS score was as follows: 0 = no pain, 1–3 = mild pain, 4–6 = moderate pain, 7–10 = severe pain.

### Statistical analysis

SPSS 23.0 software was used for statistical descriptive analysis, the enumeration data were presented as mean ± standard deviation. An independent sample *t*-test was used to compare the data between the two groups. Kruskal Wallis test was used for continuous variables when comparing three or two treatment groups, respectively. *P* < 0.05 was considered statistically significant.

## Results

### Characteristics of the patients

A total of 48 subjects were included in this study, including 33 patients in PSP group and 15 patients in the LP group. As shown in Table 1, the age of patients in PSP group was 38.18 ± 7.73 years old, and 37.33 ± 7.49 in LP group. Besides, basic indicators including BMI, volume of myoma and delivery mode such as undelivered, vaginal delivery, cesarean section, and vaginal delivery & cesarean section were also evaluated, and the statistical results showed that no significant differences were found in baseline data including age, BMI, volume of myoma and delivery mode in two groups (all *P* > 0.05) (Table 1).

**Table 1:** Baseline information of patients

Characteristic	prone position (*n* = 33)	lithotomy position (*n* = 15)	*P*-value	Statistical method
Age (year)	38.18 ± 7.73	37.33 ± 7.49	0.724	*t*-test
BMI (kg/m^2^)	21.85 ± 4.35	22.25 ± 2.99	0.991	Kruskal-Wallis test
Volume of myoma (cm^3^)	92.61 ± 63.23	92.37 ± 61.64	0.833	Kruskal-Wallis test
Delivery mode				
Undelivered	6 (18.18%)	3 (20%)	0.387	Chi-squared test
Vaginal delivery	13 (39.39%)	7 (46.67%)		
Cesarean section	14 (42.42)	4 (26.67%)		
Vaginal delivery & Cesarean section	0	1 (6.67%)		

Note: BMI, body mass index

### Comparison of surgery outcome indicators between both PSP group and LP group

To assess the difference in operation outcomes in both patient groups, we analyzed and compared the indicators including intraoperative blood loss, hemoglobin values before and 72 h after operation and VAS score. The results indicated that the intraoperative blood loss exhibited a mean of 61.21 ± 68.59 ml in the PSP group, and 88.00 ± 111.11 ml in the LP group. Regarding hemoglobin changes before and 72 h after the operation, the PSP group displayed a mean of 17.09 ± 10.82 g/dl, while the LP group showed a mean of 19.80 ± 10.96 g/dl. The VAS scores were measured at two time points: 6 h and 24 h postoperatively. At 6 h, the VAS score was 0.85 ± 0.508 in the PSP group and 0.73 ± 0.59 in the LP group. At 24 h, the scores were 0.91 ± 0.536 in the PSP group and 0.82 ± 0.71 in the LP group. Further statistical results suggested that there were no significant differences in each indicator between these two position groups (all *P* > 0.05) (Table 2).

**Table 2:** Outcome measures

Characteristic	Prone position (*n* = 33)	Lithotomy position (*n* = 15)	*P*-value	Statistical method
Intraoperative blood loss (ml)	61.21 ± 68.59	88.00 ± 111.11	0.592	Kruskal-Wallis test
Hemoglobin changes before and 72 h after operation (g/dl)	17.09 ± 10.82	19.80 ± 10.96	0.427	*t*-test
VAS score	0.85 ± 0.508	0.73 ± 0.59	0.46	Kruskal-Wallis test
Hospital length (day)	3.97 ± 2.33	4.33 ± 1.63	0.144	Kruskal-Wallis test
Operation time (minute)	93.39 ± 28.44	112.67 ± 31.57	0.041	*t*-test
Operation preparation time (minute)	21.64 ± 7.94	15.80 ± 5.82	0.016	Kruskal-Wallis test

Note: VAS, visual analog scale

### Comparison of operation-related period indicators in between PSP group and LP group

We further analyzed the operation-related period indicators between the two groups, such as hospital length, operation time and operation preparation time. As represented in Table 2, hospital length of stay was observed to be 3.97 ± 2.33 days in the PSP group and 4.33 ± 1.63 days in the LP group without significant difference. However, we found that the operation time in PSP group (93.39 ± 28.44 min) was significantly short compared with LP group (112.67 ± 31.57 min) (*P*<0.05). Besides, in terms of operation preparation time, the PSP group had a mean time of 21.64 ± 7.94 min, which was longer than LP group (15.80 ± 5.82 min) (*P*<0.05).

## Discussion

In this study, we investigated the efficiency and outcomes between PSP and LP groups in posterior uterine myomectomy by vNOTES. We analyzed a series of important baseline characteristics and outcome parameters, and the results indicated that PSP group has significantly shorter operation time and longer operation preparation time, when compared with LP group. No significant differences were found in other indicators between the two patient groups. Moreover, there were no unplanned readmissions in the present, and no severe complications such as massive hemorrhage, postoperative infection, or surgical injury occurred in both groups. Our findings suggest that altering the body position and increasing the surgical operating space can effectively reduce the duration of the procedure, which is in line with the concept that optimizing patient positioning can lead to enhanced surgical efficiency and decreased operation time.

The concept of NOTE was first introduced by Kalloo et al. [1] in 2004, revolutionized surgical techniques by utilizing natural orifices as access routes to the peritoneal cavity. Previous studies have compared the feasibility and safety of intraperitoneal surgery through the stomach, anus, urethra, and vagina, and compared the route of vaginal access to the pelvic and abdominal cavity is less complex than [12, 13, 14]. Thus, the transvaginal natural orifice surgical approach is used in various surgical procedures, including appendectomy [15], cholecystectomy [16], and sigmoid colectomy [17]. Since its inception, this approach has been embraced and adapted for various surgical procedures, with transvaginal NOTE (namely vNOTES) being particularly relevant in gynecology. In 2012, Ahn et al. [18] first published on the safety and efficacy of NOTE in gynecological surgery. Safe and effective implementation of the vNOTE procedure in urology leads to more options for gynecologic surgical approaches [19].

Since the safety and feasibility of vNOTES for myomectomy of the posterior wall of the uterus have been demonstrated in our previous studies [11], we have tried to avoid the use of the same patient data in the present study. Currently, apart from our study, there are no relevant studies on myomectomy of the posterior uterine wall in a prone split-leg position. Here, the present study contributes to the growing body of knowledge surrounding vNOTES. By establishing a surgical approach through the posterior vaginal fornix and adopting the split-leg position, we effectively reposition the myoma, enhancing surgical visibility and decreasing procedural complexities. Moreover, this approach reduces the need for multiple surgical assistants, thus streamlining the surgical process.

Strengths of the prone split-leg position include enhanced surgical visibility and increased manipulation space, which can significantly shorten operation time. This positioning approach reduces the complexity of posterior uterine myomectomy by providing a better field of view, allowing for a more efficient procedure with fewer surgical assistants. However, the main limitation of this technique is the longer preoperative preparation time, which arises from the complexity of positioning the patient and requiring additional personnel for assistance. Moreover, this position may not be suitable for all patients, particularly those with comorbidities such as obesity or severe spinal deformities, where positioning may be difficult or less effective.

In support of these findings, a similar study by Kumar et al. (2022) [20] compared the lithotomy and prone positions in perianal surgery. Their study demonstrated that the prone position offered significantly better ergonomics, reduced physical and mental stress for the operating team, and improved exposure of the surgical site compared to the lithotomy position. These results align with our study’s findings, which suggest that optimizing surgical positioning can improve visibility, reduce complexity, and enhance surgical efficiency.

There existed a noticeable distinction in the preoperative preparation time between the two studied groups, which was significantly longer in PSP group than that of LP group. For the preoperative preparation of group PSP, the patient was placed in the prone split-leg position after tracheal intubation [21, 22]. During this process, an anesthesiologist undertook the safeguarding of the patient’s head and tracheal intubation, while an additional team of 2–4 preparatory personnel facilitated a 180-degree rotation of the patient. This procedural step contributed to an extension of the preoperative preparation time, and the augmentation of preparatory personnel numbers was noted, particularly in scenarios of higher patient body mass index. In contrast, the LP group required a reduced number of 1–2 preparatory personnel to achieve the appropriate lithotomy position following endotracheal intubation anesthesia. To solve this problem, we sought to invent an automated device that could flip the patient through the device into the prone split-leg position, and then the operation preparation personnel placed the patient in the appropriate prone split-leg position, thus reducing the preoperative preparation time for the prone split-leg position.

**Fig. 4:**
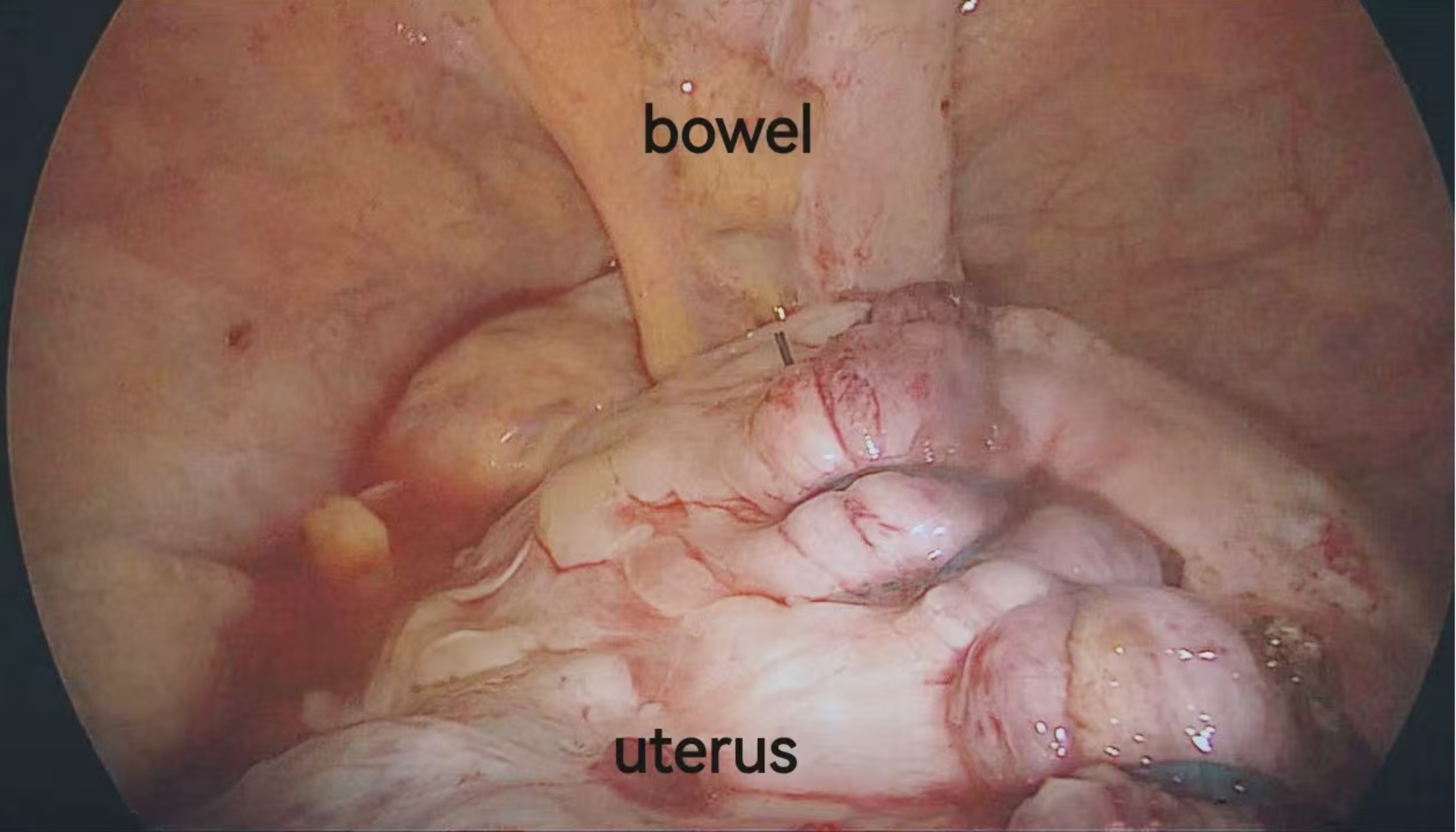
Repair of the uterine defect. This image shows the repair of the uterine defect after myomectomy, with fine suturing ensuring the integrity of the uterine wall and minimizing postoperative complications

From January 2019 to August 2022, our hospital successfully applied vNOTES in more than 3000 cases. By establishing the surgical approach through the posterior vaginal fornix and placing the patient in the prone split-leg position, we were able to transform the posterior myoma into an “anterior” position, which shortens the operation time, increases the surgical field exposure, reduces the difficulty of the procedure, and minimizes the patient’s anesthesia time. Moreover, while the application of LP for myomectomy typically requires a larger number of surgical assistants due to the complexities of the procedure, the prone split-leg position simplifies the process, requiring only one main surgical knife and one mirror holder, thus reducing the investment of surgical staff and improving the overall efficiency of surgery. The repair of the uterine defect after myomectomy is a crucial step in the procedure. Figure 4 illustrates this phase, where the fine suturing technique ensures the integrity of the uterine wall, minimizing potential postoperative complications and facilitating recovery. These findings highlight the advantages of the prone split-leg position in improving the efficiency, safety, and outcomes of posterior uterine myomectomy.

## Conclusions

Collectively, our study compared the difference of the efficiency and outcomes between PSP and LP groups in posterior uterine myomectomy by vNOTES. Our results underscore the transformative benefits derived from the surgical strategy centered on the posterior vaginal fornix approach and the adoption of the prone split-leg position. We suggested that a prone split-leg position may be considered as a helpful surgical position for myomectomy. However, it is necessary to continue to expand the sample size and conduct a comparative study with more dimensions to compare the differences between these two positions due to the small sample size in our future work.

## Electronic supplementary material

Below is the link to the electronic supplementary material.

Supplementary Material 1

## Data Availability

The data that support the findings of this study are available from the corresponding author upon reasonable request.

## Abbreviations

vNOTES: transvaginal natural orifice transluminal endoscopic surgery
NOTES: Natural orifice transluminal endoscopic surgery
PSP group: prone split-leg position group
LP group: lithotomy position group

## References

[1] Kalloo AN, Singh VK, Jagannath SB, Niiyama H, Hill SL, Vaughn CA, et al. Flexible transgastric peritoneoscopy: a novel approach to diagnostic and therapeutic interventions in the peritoneal cavity. Gastrointest Endosc. 2004;60:114–7. 15229442 10.1016/s0016-5107(04)01309-4

[2] Lee CL, Wu KY, Su H, Ueng SH, Yen CF. Transvaginal natural-orifice transluminal endoscopic surgery (NOTES) in adnexal procedures. J Minim Invasive Gynecol. 2012;19:509–13. 22425142 10.1016/j.jmig.2012.02.005

[3] Wilson M, Ramage L, Yoong W, Swinhoe J. Femoral neuropathy after vaginal surgery: a complication of the lithotomy position. J Obstet Gynaecology: J Inst Obstet Gynecol. 2011;31:90–1. 10.3109/01443615.2010.528082 21281008

[4] Bauer EC, Koch N, Janni W, Bender HG, Fleisch MC. Compartment syndrome after gynecologic operations: evidence from case reports and reviews. Eur J Obstet Gynecol Reprod Biol. 2014;173:7–12. 24290432 10.1016/j.ejogrb.2013.10.034

[5] Yassa M, Kaya C, Kalafat E, Tekin AB, Karakas S, Mutlu MA et al. The comparison of transvaginal natural orifice transluminal endoscopic surgery and conventional laparoscopy in opportunistic bilateral salpingectomy for permanent female sterilization. J Minim Invasive Gynecol. 2022;29:257– 64.e1. 10.1016/j.jmig.2021.08.009 34411729

[6] Karakaş S, Kaya C, Yildiz Ş, Alay İ, Durmuş U, Aydiner İE, et al. Comparison of vNOTES technique with conventional laparoscopy in gynecological emergency cases. Minim Invasive Ther Allied Technol. 2022;31:803–9. 35073493 10.1080/13645706.2021.2025111

[7] Kaya C, Alay I, Yildiz S, Cengiz H, Afandi X, Yasar L. The feasibility of natural orifice transluminal endoscopic surgery in gynecology practice: Single-Surgeon experience. Gynecol Minim Invasive Ther. 2020;9:69–73. 32676283 10.4103/GMIT.GMIT_84_19 PMC7354749

[8] Tekin AB, Yassa M, Kaya C, Budak D, Ilter PB, Mutlu MA, et al. Implementing the transvaginal natural orifice transluminal endoscopic surgery (vNOTES) first strategy in benign gynecological surgeries. Arch Gynecol Obstet. 2023;307:1007–13. 36445449 10.1007/s00404-022-06859-9

[9] Ketenci Gencer F, Salman S, Kumbasar S, Bacak HB, Khatib O, Kaya C, et al. Lateral suspension with V-NOTES for the treatment of pelvic organ prolapse with the Salman-Ketenci gencer technique. Int Urogynecol J. 2023;34:1583–91. 36625926 10.1007/s00192-022-05433-w

[10] Tekin AB, Yassa M, Kaya C, Birol Ilter P, Mutlu MA, Kalkan U, et al. Vaginal birth following diagnostic vNOTES and appendectomy in a pregnant woman: a case report. J Obstet Gynaecol. 2022;42:3387–9. 36018048 10.1080/01443615.2022.2114330

[11] Huang L, He L, Zhang L, Gan X, Jia J, Yang Y, et al. Application of the prone position in myomectomy by transvaginal natural orifice transluminal endoscopic surgery. Wideochirurgia I Inne Techniki maloinwazyjne = Videosurgery Other Miniinvasive Techniques. 2021;16:234–42. 33786139 10.5114/wiitm.2020.95397 PMC7991957

[12] Autorino R, Yakoubi R, White WM, Gettman M, De Sio M, Quattrone C, et al. Natural orifice transluminal endoscopic surgery (NOTES): where are we going? A bibliometric assessment. BJU Int. 2013;111:11–6. 23323699 10.1111/j.1464-410X.2012.11494.x

[13] Pearl JP, Ponsky JL. Natural orifice transluminal endoscopic surgery: past, present and future. J Minim Access Surg. 2007;3:43–6. 21124650 10.4103/0972-9941.33271 PMC2980719

[14] Pearl JP, Ponsky JL. Natural orifice translumenal endoscopic surgery: A critical review. J Gastrointest Surg. 2008;12:1293–300. 18057995 10.1007/s11605-007-0424-4

[15] Jayaraman S, Schlachta CM. Transgastric and transperineal natural orifice translumenal endoscopic surgery (NOTES) in an appendectomy test bed. Surg Innov. 2009;16:223–7. 19666934 10.1177/1553350609342076

[16] Marescaux J, Dallemagne B, Perretta S, Wattiez A, Mutter D, Coumaros D. Surgery without scars: report of transluminal cholecystectomy in a human being. Archives Surg (Chicago Ill: 1960). 2007;142:823–6. discussion 6–7. 10.1001/archsurg.142.9.823 17875836

[17] Alba Mesa F, Amaya Cortijo A, Romero Fernandez JM, Komorowski AL, Sanchez Hurtado MA, Fernandez Ortega E, et al. Transvaginal sigmoid cancer resection: first case with 12 months of follow-up–technique description. J Laparoendosc Adv Surg Tech Part A. 2012;22:587–90. 10.1089/lap.2011.0469 22690651

[18] Ahn KH, Song JY, Kim SH, Lee KW, Kim T. Transvaginal single-port natural orifice transluminal endoscopic surgery for benign uterine adnexal pathologies. J Minim Invasive Gynecol. 2012;19:631–5. 22763314 10.1016/j.jmig.2012.04.001

[19] Kaouk JH, White WM, Goel RK, Brethauer S, Crouzet S, Rackley RR, et al. NOTES transvaginal nephrectomy: first human experience. Urology. 2009;74:5–8. 19567279 10.1016/j.urology.2009.03.030

[20] Kumar P, Mishra TS, Sarthak S, Sasmal PK. Lithotomy versus prone position for perianal surgery: a randomized controlled trial. Ann Coloproctol. 2022;38:117–23. 34098632 10.3393/ac.2020.12.16 PMC9021856

[21] Zambouri A. Preoperative evaluation and Preparation for anesthesia and surgery. Hippokratia. 2007;11:13–21. 19582171 PMC2464262

[22] Keorochana G, Muljadi JA, Kongtharvonskul J. Perioperative and radiographic outcomes between Single-Position surgery (Lateral Decubitus) and Dual-Position surgery for lateral lumbar interbody fusion and percutaneous pedicle screw fixation: Meta-Analysis. World Neurosurg. 2022;165:e282–91. 35710097 10.1016/j.wneu.2022.06.029

