# Supplementary material for: A comparative study of prone split-leg position and lithotomy position in posterior uterine myomectomy by transvaginal natural orifice transluminal endoscopic surgery
Source: BMC Womens Health. 2025 Apr 12;25:177. doi: 10.1186/s12905-025-03709-z (PMC11994009; doi:10.1186/s12905-025-03709-z)
Supplement: Supplementary file 1 — Supplementary Material 1 [file 12905_2025_3709_MOESM1_ESM.pdf]

伦理审查批件

批件号：科研伦审 2020（67）号

项目名称	俯卧分腿位与膀胱截石位在经阴道自然腔道内镜手术（V-NOTES）子宫后壁肌瘤剥除术中的有效性及安全性的随机对照研究		
类别	1.医学科研（√）2.新技术新业务（ ）3.辅助生殖（ ）4.产前诊断（ ）		
审查方式	1.会议审查（ ）2.快速审查（√ ）		
主审委员	谢晓丽 李根		
项目负责人	何丽 主任医师	所属部门	妇科
**审查文件**	**复审文件：**<br>1.复审申请表<br>2.知情同意书（版本号：V2.0，版本日期：2020 年 06 月 26 日）		
根据我国相关法律、法规和规范，以及伦理原则，经本伦理委员会审查决定	同意<br>研究负责人必须严格使用经审查同意的知情同意书文本和研究方案。如伦理审查批件失效时不能完成所有的临床研究（包括统计分析），请在本批件失效前一个月，递交持续审查申请。<br>1.如研究结束并在审查有效期内，请递交结题报告；<br>2.研究中发生涉及受试者或其他人风险的任何 SAE 或非预期的不良事件，应立刻报告本伦理委员会；<br>3.任何研究方案、知情同意书的修改包括研究人员的变更，必须递交研究方案修改申请表，经伦理委员会审查获得批准后执行；<br>4.出现没有遵从方案开展研究的情况，或可能对受试者的权益/健康、以及研究的科学性造成不良影响等情况，请及时提交违背方案报告至本伦理委员会；<br>5.请按照跟踪审查的频率要求，在预计审查日期前 1 月提交跟踪审查报告；<br>6.申请人暂停或提前终止临床试验，请及时提交暂停/终止研究报告。		
伦理审查批件有效期	2020 年 6 月 28 日至<br>2022 年 6 月 28 日	跟踪审查频率	12 个月
主任委员签字	[signature]	日期	2020 年 6 月 28 日
声明：本伦理委员会的职责、人员组成、运行和记录遵循中华人民共和国卫生和计划生育委员会 2016 年颁布的《涉及人的生物医学研究伦理审查办法》和《赫尔辛基宣言》的伦理原则，并遵守中国的相关法律及法规。			
联系地址：成都市青羊区日月大道 1617 号	联系电话：028-61866015；<br>028-61866009	邮箱：fezxywb@qq.com	
